# Biomarkers of perioperative neurocognitive disorders after cardiac surgery: from mechanistic insights to clinical translation

**DOI:** 10.3389/fnins.2026.1893171

**Published:** 2026-08-14

**Authors:** Xiaojin Li, Rui Yu, Xu Ku, Kaixin Wang, Yawen Peng, Xiangdong Chen

**Affiliations:** 1 Department of Anesthesiology, Union Hospital, Tongji Medical College, Huazhong University of Science and Technology, Wuhan, China; 2 Institute of Anesthesia and Critical Care Medicine, Union Hospital, Tongji Medical College, Huazhong University of Science and Technology, Wuhan, China; 3 Key Laboratory of Anesthesiology and Resuscitation (Huazhong University of Science and Technology), Ministry of Education, Wuhan, China

**Keywords:** biomarkers, cardiac surgery, neuroinflammation, perioperative neurocognitive disorders, translation

## Abstract

Perioperative neurocognitive disorders (PND) after cardiac surgery are common complications that adversely affect postoperative recovery, functional independence, and long-term prognosis. Their pathogenesis is multifactorial, involving cerebral hypoperfusion, microembolism, CPB-related systemic inflammation, BBB disruption, oxidative stress, metabolic dysregulation, neuroimmune imbalance, and host vulnerability. This review summarizes current evidence on major biomarker categories, including neuronal and axonal injury, glial activation, BBB dysfunction, systemic and neuroinflammation, oxidative stress, neurotrophic signaling, metabolic disturbance, extracellular vesicles (EVs), microRNAs, gut-brain axis-related signals, and clinical vulnerability factors. Neurofilament light chain (NfL), glial fibrillary acidic protein (GFAP), and several inflammatory markers have the most direct clinical evidence, although none has shown sufficient standalone performance for routine use. EVs, microRNAs, metabolomic profiles, pro-resolving mediators, autonomic-neuroimmune indicators, and microbiome-derived signals remain exploratory. However, most candidate biomarkers show limited discriminatory power when used alone, and few have demonstrated incremental value beyond established perioperative risk factors, including age, frailty, preoperative cognitive impairment, hemodynamic instability, and CPB-related variables. Biomarker-informed intervention studies remain predominantly preclinical or hypothesis-generating. Future progress will require standardized outcome definitions, harmonized sampling strategies, multicenter validation, and temporally structured multimodal models tailored to specific clinical purposes, including preoperative risk stratification, early postoperative brain injury monitoring, prognostic assessment, and treatment-response evaluation.

## Graphical Abstract

**Figure:**
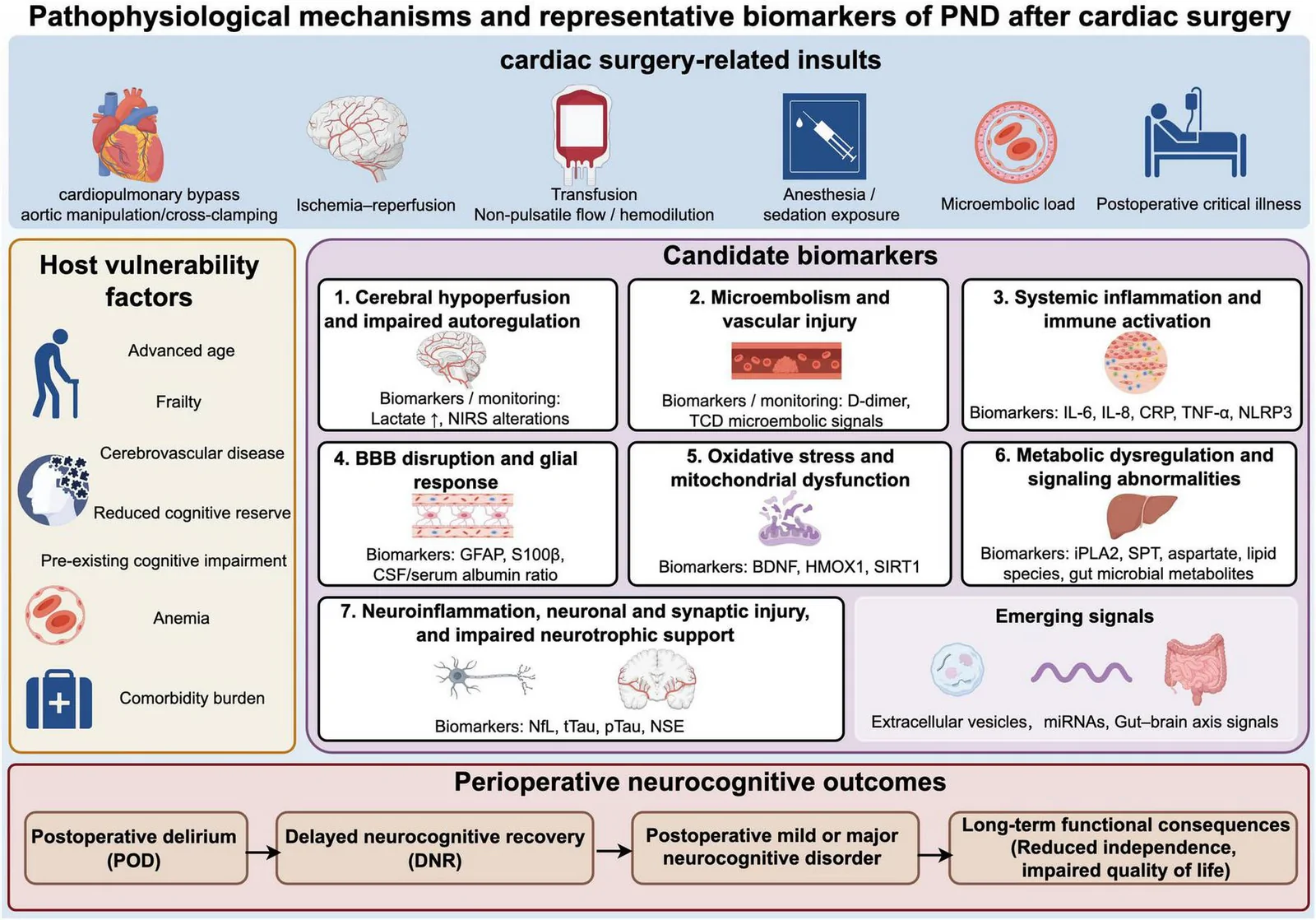
Pathophysiological mechanisms and representative biomarkers of perioperative neurocognitive disorders (PND) after cardiac surgery. The graphical abstract summarizes cardiac surgery-related insults, host vulnerability factors, major pathophysiological pathways, representative biomarker domains, emerging exploratory signals, and perioperative neurocognitive outcomes.

## 1 Introduction

Perioperative neurocognitive disorders (PND) encompass several clinically distinct outcomes after surgery. The 2018 nomenclature framework includes pre-existing neurocognitive disorder, postoperative delirium (POD), delayed neurocognitive recovery (DNR), and postoperative mild or major neurocognitive disorder, while separating acute delirium from objectively measured cognitive decline (Evered et al., 2018). Earlier cardiac-surgery studies generally used postoperative cognitive dysfunction as an umbrella term, which partly explains the heterogeneity of the literature (Gao et al., 2005; Goto and Maekawa, 2014; Yuan and Lin, 2019). This terminology has been further reinforced in subsequent narrative reviews, which emphasize that perioperative cognitive outcomes should not be viewed merely as research endpoints, but as patient-centered complications associated with mortality, loss of independence, impaired functional recovery, and an increased risk of long-term cognitive deterioration (Evered L. et al., 2022).

Cardiac surgery represents a particularly high-risk setting for PND because it involves multiple perioperative insults that may converge on the vulnerable brain. Cardiopulmonary bypass (CPB), aortic cross-clamping, non-pulsatile flow, hemodilution, hypothermia, microembolism, systemic inflammatory activation, ischemia-reperfusion injury, blood-brain barrier (BBB) disruption, and exposure to postoperative critical care may all contribute to neurocognitive vulnerability (Siepe et al., 2011; Toth et al., 2013; Aldana et al., 2014; Vlisides et al., 2019). The contribution of any single exposure is difficult to isolate. A systematic review of intraoperative hypotension in cardiac surgery emphasized that safe blood pressure targets remain uncertain, and that the relationship among mean arterial pressure, cerebral autoregulation, and postoperative neurocognitive outcomes are highly heterogeneous across studies (Czok et al., 2021). Anesthetic exposure is similarly entangled with the effects of surgery and critical care (Evered and Scott, 2020). Consistent with this concept, the ENGAGES-Canada randomized clinical trial showed that electroencephalography (EEG)-guided anesthetic administration reduced volatile anesthetic exposure and EEG suppression time, but did not reduce postoperative delirium after cardiac surgery in older adults (Deschamps et al., 2024). Collectively, these findings indicate that PND after cardiac surgery should not be interpreted as the consequence of a single intraoperative variable, but rather as the result of dynamic interactions among cerebral perfusion, inflammatory burden, pre-existing vulnerability, and postoperative recovery trajectories.

Current diagnosis and assessment of PND still rely primarily on clinical manifestations, delirium screening tools, and neuropsychological testing. Although indispensable, these approaches have several important limitations (Bilotta et al., 2014; Majewski et al., 2020). POD is usually assessed during the early postoperative period using bedside tools (Veliz-Reissmüller et al., 2007; Chen et al., 2021), whereas DNR and postoperative neurocognitive disorder require longitudinal cognitive testing and appropriate baseline assessment (Kassie et al., 2017; Nomura et al., 2019). Differences in diagnostic criteria, neuropsychological test batteries, follow-up duration, and statistical definitions of cognitive decline have contributed substantially to heterogeneity across studies. In cardiac surgery, this heterogeneity is further amplified by variability in surgical procedure, CPB exposure, cerebral perfusion strategy, postoperative intensive care management, and comorbidity burden (Akhtar et al., 2021). Therefore, objective biomarkers are increasingly being investigated as complementary tools for early risk identification, mechanistic phenotyping, dynamic monitoring, and prognostic assessment.

This review summarizes current evidence on biomarkers associated with PND following cardiac surgery, with particular attention to procedure-specific exposures, sampling time, endpoint definition, and clinical interpretability. Terminology and timing are kept separate throughout. POD is an acute, fluctuating disturbance of attention and awareness in the early postoperative period. DNR denotes objectively measured decline within 30 days, whereas postoperative mild or major neurocognitive disorder is diagnosed from 30 days to 12 months (Evered et al., 2018). A biomarker measured before surgery, immediately after CPB, or 1 week later cannot be assumed to represent the same biological process, even when each measurement is associated with an outcome labeled PND. The evidence covers neuronal and axonal injury, glial and BBB dysfunction, inflammation, oxidative and neurotrophic pathways, metabolic disturbance, EVs, and gut-brain axis dysregulation. (Feldman et al., 2013; Liu et al., 2021; Coverdale et al., 2025). However, their clinical roles remain incompletely defined, therefore, the discussion separates biological association from adjusted prediction, added value beyond clinical variables, and evidence that a result could alter management.

To provide an integrated overview, the Graphical Abstract summarizes how cardiac surgery-related insults and host vulnerability factors may converge on neurovascular dysfunction, systemic and central inflammation, oxidative stress, metabolic dysregulation, and neuronal/synaptic injury, and maps representative biomarker categories onto these processes.

## 2 Major biomarker categories and their clinical relevance

### 2.1 Neuronal and axonal injury biomarkers

Perioperative neurocognitive abnormalities following cardiac surgery are frequently associated with cerebral hypoperfusion, microembolism, and CPB-related physiological stress. Accordingly, biomarkers of neuronal and axonal injury have been among the most extensively investigated indicators in this field (Grocott et al., 2005; Bruggemans, 2013; Goto and Maekawa, 2014). Release kinetics differ substantially between markers, and studies have not used consistent sampling windows or neurocognitive endpoints. A postoperative rise may therefore indicate acute injury, delayed axonal degeneration, or pre-existing vulnerability, depending on the marker and time point.

Among axonal injury biomarkers, neurofilament light chain (NfL) has received particular attention because of its relatively specific association with neuroaxonal damage. In patients undergoing cardiac surgery, increased NfL levels have been linked to postoperative cognitive impairment, especially when measured at later postoperative time points such as discharge or postoperative day 7 (Alifier et al., 2020; Wiberg et al., 2022). The signal is not exclusively postoperative. In a nested study of 175 patients, higher preoperative NfL was associated with delirium and delirium severity, whereas the early perioperative change was not (Brown et al., 2024). Baseline NfL may therefore capture pre-existing cerebral vulnerability in addition to perioperative injury. A systematic review and meta-analysis found an overall association between NfL and PND but also substantial variation in endpoints, sampling schedules, and assays (Chen et al., 2026). No cardiac-surgery-specific threshold has been validated, and its added value over clinical risk factors remains uncertain.

Tau-related biomarkers appear to reflect a different dimension of perioperative brain injury. Compared with NfL, plasma tau may rise earlier during the perioperative period, suggesting a closer relationship with acute neuronal stress or injury (Alifier et al., 2020). Changes in total tau and phosphorylated tau-181 have also been associated with postoperative neurocognitive decline, recovery trajectories, and delirium-related outcomes, supporting their potential value as markers of acute neuronal perturbation (McKay et al., 2022; Kaushik et al., 2025). However, the interpretation of perioperative tau elevation requires caution. In this setting, increased tau should not be directly equated with Alzheimer-type pathology, because transient neuronal stress, surgical inflammation, BBB dysfunction, and perioperative physiological disturbances may all influence tau release. At present, tau is therefore better regarded as a process-oriented biomarker reflecting acute neuronal stress or injury, rather than as a mature standalone predictor of PND.

Neuron-specific enolase (NSE), a marker of neuronal cytoplasmic injury, has also been widely studied, but its performance in cardiac surgery has been inconsistent. Some studies suggest that NSE may indicate perioperative brain injury, whereas its ability to identify postoperative cognitive impairment appears limited and may be inferior to that of other markers such as S100β (Silva et al., 2016). Other evidence has shown no significant association between NSE and postoperative cognitive impairment at discharge (Wiberg et al., 2022). These discrepancies likely reflect not only biological limitations of NSE itself, but also methodological and perioperative confounders, including sampling time, CPB-associated hemolysis, blood transfusion, systemic inflammation, and heterogeneity in cognitive outcome definitions (Hassani Ahangar et al., 2025). This is particularly important in cardiac surgery, where extracerebral sources and procedure-related artifacts may obscure the relationship between circulating NSE levels and true neuronal injury. Overall, biomarkers of neuronal and axonal injury may be more useful for dynamic monitoring, mechanistic interpretation, and risk stratification than as precise standalone predictive tools.

### 2.2 Glial responses and blood-brain barrier dysfunction biomarkers

Following neuronal and axonal injury biomarkers, glial and blood-brain barrier (BBB)-related biomarkers provide a complementary perspective by reflecting perioperative changes in the intracerebral microenvironment. Cardiopulmonary bypass, systemic inflammation, and perfusion abnormalities do not necessarily lead to immediate and significant neuronal necrosis; rather, they are likely to first trigger astrocyte activation and increase BBB permeability (Grocott et al., 2005; Bruggemans, 2013). This stage may represent a transition from peripheral surgical stress to central neurovascular unit dysfunction, which is highly relevant to postoperative delirium and delayed neurocognitive recovery.

Glial fibrillary acidic protein (GFAP) is a representative marker of astrocytes. Evidence from cerebrospinal fluid studies has shown that GFAP levels increase after CPB, accompanied by an elevation in the cerebrospinal fluid/serum albumin ratio, suggesting that glial injury and BBB dysfunction may occur in parallel (Reinsfelt et al., 2012). Postoperative increases in circulating GFAP have also been associated with cognitive impairment at discharge, although its predictive performance appears only moderate (Wiberg et al., 2022). In a small exploratory study of 30 patients undergoing off-pump CABG, a panel combining miR-21-5p, GFAP, and neuroserpin showed a high apparent area under the curve for early cognitive dysfunction; the limited sample, low event count, and absence of external validation make overfitting a substantial concern (Szwed et al., 2021). Taken together, current findings support the interpretation of GFAP as a biomarker of perioperative central nervous system injury rather than as a marker specific to a single neurocognitive phenotype. Therefore, GFAP may be more useful as part of a multimodal brain injury panel than as an isolated diagnostic biomarker for PND.

S100 calcium-binding protein B (S100β) is commonly regarded as a surrogate biomarker of astrocytic injury and BBB permeability. In some studies, S100β appears to have greater utility than neuron-specific enolase (NSE) for identifying postoperative cognitive impairment (Silva et al., 2016). However, both NSE and S100β remain classic but controversial biomarkers in cardiac surgery-related neurocognitive research, largely because their perioperative interpretation is vulnerable to biological and methodological confounders (Ji and Li, 2022). In particular, S100β is highly dependent on the sampling window and may be influenced by surgical trauma, extracerebral release, and peripheral tissue injury, which limits its clinical specificity despite its research value (Hassani Ahangar et al., 2025). The cerebrospinal fluid/serum albumin ratio has clearer pathophysiological relevance for assessing BBB integrity, but the invasiveness and limited feasibility of cerebrospinal fluid sampling restrict its application in routine clinical screening (Reinsfelt et al., 2012). Overall, glial and BBB-related biomarkers may be most informative for identifying the shift from structural neuronal injury toward neurovascular and glial dysfunction, rather than serving as standalone diagnostic tools for PND.

### 2.3 Systemic inflammation and neuroinflammation-related biomarkers

Inflammation biomarkers provide another important but conceptually distinct dimension of biomarker assessment in PND after cardiac surgery. Unlike neuronal, axonal, glial, or blood-brain barrier-related markers, inflammatory biomarkers usually reflect the magnitude, trajectory, and resolution of the perioperative systemic-central inflammatory response rather than brain injury (Peng et al., 2013; Evered L. A. et al., 2022). Greater relevance may lie in an excessive or persistent response that coincides with BBB dysfunction, glial activation, or neuronal injury.

Among conventional inflammatory cytokines, interleukin-6 (IL-6) has been one of the most extensively investigated. Meta-analytic evidence across surgical populations supports an association between higher perioperative IL-6 and postoperative cognitive decline, but study designs and outcome definitions vary widely (Peng et al., 2013). However, single postoperative measurements may provide only limited information, as inflammatory trajectories appear to be more informative than isolated values at a single time point (Evered L. A. et al., 2022). Interleukin-8 (IL-8), which is more closely related to inflammatory cell recruitment, may offer complementary information about central inflammatory activity. In cerebrospinal fluid studies after thoracic aortic surgery have also shown postoperative changes in IL-8 and other cytokines, documenting central inflammatory activation without establishing independent prediction of a neurocognitive endpoint (Pereira et al., 2022). C-reactive protein (CRP), as a routinely available acute-phase reactant, has practical advantages because of its ease of measurement. Nevertheless, although CRP has been associated with early postoperative cognitive abnormalities, its independent contribution appears limited, and its lack of neurological specificity restricts its value as a standalone biomarker (Goettel et al., 2017; Majewski et al., 2020).

In addition to conventional cytokines, inflammasome-related markers may provide a more pathway-specific inflammatory signal. In a small observational cardiac-surgery cohort, serum NLRP3, caspase-1, IL-1β, and IL-18 increased after surgery, and end-of-surgery NLRP3 was independently associated with early PND (Ma et al., 2022). These findings suggest that NLRP3 inflammasome activation may represent a mechanistic link between perioperative innate immune activation and early postoperative cognitive decline. However, the current evidence remains limited and largely observational. Therefore, replication in larger cohorts, with standardized cognitive endpoints and external validation, is required before NLRP3 can be regarded as more than an exploratory marker.

Another emerging concept is that postoperative neuroinflammation may result not only from excessive inflammatory activation but also from impaired resolution of inflammation. Specialized pro-resolving mediators, including resolvins, maresins, protectins, and lipoxins, have been proposed to regulate postoperative neuroinflammation by modulating microglial activation, astrocyte reactivity, macrophage infiltration, and blood-brain barrier integrity (Yang and Terrando, 2019). Although these mediators remain largely preclinical in the PND field, they broaden the biomarker framework from inflammatory activation to inflammatory resolution.

### 2.4 Oxidative stress, neurotrophic factors, and endogenous protective pathways

Brain-derived neurotrophic factor (BDNF) is a representative molecule among this class of markers. In a prospective pilot study of coronary artery bypass surgery, lower BDNF during aortic cross-clamping was associated with early PND (Miniksar et al., 2021). Persistent postoperative reductions in BDNF have also been linked to more pronounced inflammatory and oxidative stress abnormalities, indicating that loss of neurotrophic support may occur alongside broader disturbances in perioperative homeostasis (Miceli et al., 2025). These observational data are compatible with impaired neurotrophic support, but they do not show that BDNF is an independent predictor or a direct measure of neuronal injury.

Antioxidant and stress-response pathways provide another mechanistically relevant biomarker domain. Heme oxygenase-1 (HMOX1), together with related oxidative stress markers, may reflect the capacity to counterbalance perioperative oxidative and inflammatory injury. In the CABG cohort, patients with early perioperative neurocognitive dysfunction have been reported to show decreased HMOX1 levels during follow-up, accompanied by increased lectin-like oxidized low-density lipoprotein receptor-1 (LOX-1) levels and persistent inflammatory activation (Miceli et al., 2025). This pattern suggests that insufficient antioxidant defense, rather than oxidative stress alone, may contribute to sustained cognitive vulnerability after cardiac surgery.

Sirtuin 1 (SIRT1) is an endogenous protective molecule that has attracted increasing attention because of its links to inflammation, oxidative stress, and neuronal resilience. In an observational cohort of older patients undergoing cardiac surgery with cardiopulmonary bypass and aortic cross-clamping, lower early postoperative serum SIRT1 concentrations were associated with postoperative cognitive dysfunction and were inversely correlated with neuron-specific enolase (NSE) and S100β levels (Shi et al., 2024). Experimental evidence further suggests that SIRT1 activation may attenuate neuroinflammation by inhibiting the Toll-like receptor 4/nuclear factor-κB (NF-κB) pathway and improving learning and memory performance (Shi et al., 2020). Complementary evidence from a rat cardiopulmonary bypass model showed that combined remimazolam and andrographolide treatment was associated with activation of the AMPK/SIRT1 pathway, reduced inflammatory responses and hippocampal apoptosis, and improved cognitive performance (Chen et al., 2025). These findings identify SIRT1-related signaling as a mechanistically relevant pathway in experimental and clinical cardiac surgery settings, but they do not establish SIRT1-targeted treatment as effective in patients.

At present, these markers are most useful for mechanistic stratification. Markers of oxidative stress, neurotrophic support, and endogenous defense may reflect biological reserve or failure of adaptation rather than established structural injury (Shi et al., 2020, 2024; Miniksar et al., 2021). This distinction is potentially useful, but the human evidence is limited. Small cohorts, variable sampling schedules, and uncertain assay thresholds prevent confident assessment of predictive performance. Overall, their predictive performance, assay feasibility, and clinical applicability in human cardiac surgery populations require further validation in well-designed prospective studies.

### 2.5 Metabolomics-related candidate biomarkers

Metabolomics-related biomarkers represent a new field that has emerged in recent years; what sets them apart is their ability to provide a more holistic view of energy metabolism, lipid remodeling, and cellular homeostasis imbalances. The reported signals involved phosphatidylinositol, glycerophospholipid, sphingomyelin, and related lipid species, although the individual metabolites and their discriminatory performance varied across studies (Huang et al., 2022; Han et al., 2023; Chen et al., 2023). These differences may reflect variation in analytical platforms, sampling time, cognitive endpoints, and feature-selection methods. Because samples were generally collected after surgery, the resulting models are better interpreted as tools for early postoperative risk classification than as predictors of preoperative susceptibility.

Experimental findings provide some biological context for the clinical lipidomic signals. In a rat model of cardiopulmonary bypass, there was a decrease in calcium-independent phospholipase A2 (iPLA2) and an increase in serine palmitoyl transferase (SPT) in the hippocampus, accompanied by synaptic structural abnormalities and impaired cognitive function (Lei et al., 2024). Multi-omics evidence has also revealed coordinated alterations across fecal, serum, and hippocampal profiles, suggesting that peripheral metabolic changes may be associated with central protein homeostasis and neurocognitive vulnerability (Qu et al., 2024). In addition, changes in hippocampal aspartate levels have been linked to the severity of cognitive impairment, further supporting the relevance of amino acid metabolism in postoperative neurocognitive dysfunction (Hu et al., 2014).

Current evidence therefore supports perioperative lipid dysregulation as a reproducible biological feature of cardiac surgery-associated neurocognitive injury, but not yet as a clinically validated biomarker domain. Although these findings broaden the biomarker framework from isolated molecular injury signals to systemic metabolic phenotyping, most evidence still comes from animal studies or small exploratory clinical cohorts. Therefore, metabolomics-related markers are currently best regarded as research candidate biomarkers for mechanistic discovery and risk phenotyping, rather than as tools ready for direct incorporation into routine clinical screening.

### 2.6 Peripheral cholinergic-related candidate biomarkers

The cholinergic pathway has long been associated with delirium and postoperative cognitive dysfunction. Accordingly, several peripheral markers related to cholinergic function have been investigated as candidate biomarkers (Plaschke et al., 2013). However, their interpretation remains challenging because peripheral cholinergic activity may not reliably reflect central cholinergic neurotransmission or brain-specific injury.

In a prospective single-center study of 114 cardiac-surgery patients, lower preoperative acetylcholinesterase (AChE) activity was independently associated with POD, while a greater postoperative reduction in butyrylcholinesterase (BChE) activity was observed in patients who became delirious (Adam et al., 2020). By contrast, earlier work in a mixed cardiosurgical cohort found no clear separation in perioperative AChE or BChE activity between patients with and without delirium, illustrating the sensitivity of these measurements to study population, operative procedure, sampling schedule, and delirium ascertainment (John et al., 2017). More procedure-specific data have emerged from patients undergoing elective on-pump coronary artery bypass grafting. In a prospective observational study of 93 patients, the decline in BChE activity from baseline to postoperative day 1 was greater in those who developed POD, whereas AChE activity showed little discriminatory value. A reduction in BChE activity of at least 32% yielded only moderate apparent discrimination and was not externally validated (Zajonz et al., 2023). A subsequent cohort including cardiothoracic surgery and percutaneous valve procedures likewise found associations between lower perioperative BChE activity and POD in unadjusted analyses, but the relationship weakened after adjustment for age, comorbidities, type of surgery, and blood transfusion (Schlake et al., 2024).

These findings suggest that cholinergic-related markers may reflect delirium vulnerability, systemic physiological reserve, or perioperative illness burden rather than specific structural brain injury. Overall, although cholinergic biomarkers are mechanistically relevant, current evidence supports their use primarily as exploratory or auxiliary variables rather than established clinical biomarkers.

### 2.7 Host vulnerability factors and integrated biomarker models

Frailty, mild preoperative cognitive decline, anemia, and related clinical variables are not molecular biomarkers in the strict sense, but they are essential contextual factors for interpreting biomarker data in cardiac surgery-related PND (Kok et al., 2017; Ke et al., 2025). Their inclusion is important because molecular signals cannot be meaningfully interpreted without considering baseline brain vulnerability, physiological reserve, and perioperative risk exposure. In many predictive models, these clinical factors are incorporated alongside molecular biomarkers and may substantially influence the apparent performance and clinical relevance of biomarker-based prediction (Cai et al., 2022; Chen et al., 2021; Shayan et al., 2022).

Frailty is one of the most consistently recognized host vulnerability factors. Patients with frailty or pre-frailty have an increased risk of postoperative delirium after cardiac surgery (Nomura et al., 2019). A meta-analysis including 16 cohort studies and 4,805 older patients further showed that preoperative frailty is independently associated with PND, with a more pronounced association in cardiac surgery than in non-cardiac surgery populations (Zhao et al., 2024). Preoperative cognitive status is another critical determinant. Mild preoperative cognitive decline has been closely associated with postoperative delirium and other adverse neurocognitive outcomes, indicating that postoperative PND often reflects not only perioperative injury but also pre-existing cognitive vulnerability (Veliz-Reissmüller et al., 2007; Chen et al., 2021). A separate meta-analysis linked preoperative cognitive impairment with adverse outcomes after cardiac surgery (Au et al., 2023). Hematologic reserve may also be relevant, as preoperative anemia and lower minimum hemoglobin levels during CPB have been associated with postoperative changes in specific cognitive domains (Shayan et al., 2022).

These findings highlight an important methodological issue: biomarker studies that do not adequately account for host vulnerability may overestimate the independent value of molecular markers. Integrated models should therefore distinguish between biomarkers that reflect perioperative injury, markers that indicate inflammatory or metabolic responses, and clinical variables that define baseline susceptibility. Only through such integration can biomarker-based frameworks move beyond statistical association toward clinically meaningful risk stratification.

### 2.8 Emerging multidimensional biomarkers and neuroimmune regulatory signals

In addition to traditional markers associated with brain injury, inflammation, and oxidative stress, EVs, noncoding RNAs, microbiome-related signals, and autonomic measures extend the biomarker field beyond conventional proteins (Carrozzo et al., 2025; Jian et al., 2025). They may capture cell of origin, regulatory cargo, host susceptibility, or inter-organ signaling. Cardiac-surgery-specific evidence is sparse, however, and analytical standardization remains a major obstacle.

EVs are of particular interest because they may function not only as circulating biomarkers but also as mediators of intercellular communication. Clinical evidence suggests that circulating EV profiles may be associated with perioperative complications, including adverse neurological outcomes, after cardiac surgery (Carrozzo et al., 2025; Jian et al., 2025). Mechanistic studies further indicate that EVs can participate in post-injury regulatory responses, supporting their potential relevance to neuroimmune and vascular signaling after cardiac surgery (Barile et al., 2014; Schmitz et al., 2025). However, EV-based biomarkers remain technically challenging because of variability in isolation methods, vesicle characterization, cargo quantification, and platform standardization.

Circulating noncoding RNAs represent another promising but still exploratory biomarker class (Min and Chan, 2015). In a study of 30 patients undergoing off-pump CABG, the combination of microRNA-21-5p with GFAP and neuroserpin (NSP) has shown better performance than individual markers for identifying early postoperative cognitive dysfunction (Szwed et al., 2021). Other evidence has linked increased serum lncRNA-MYL2-2 and decreased miR-124-3p with postoperative cognitive decline, suggesting that noncoding RNA signatures may capture regulatory changes not reflected by conventional protein biomarkers (Xiao et al., 2021). These findings suggest that noncoding RNAs may capture perioperative regulatory responses not reflected by conventional proteins, but they remain discovery signals rather than validated biomarkers.

Genetic and epigenetic biomarkers provide a different type of information, reflecting long-term susceptibility rather than acute perioperative injury. Recent narrative evidence suggests that PND may be influenced by genetic traits related to neurodegeneration, circadian rhythm regulation, inflammation, and immune responses. Epigenetic mechanisms, including DNA methylation, histone modifications, and noncoding RNA activity, may further link perioperative stressors with immune activation, neurotransmission, and neuronal vulnerability (Thedim et al., 2026). These approaches may eventually support precision risk stratification and individualized perioperative brain health management. However, their clinical implementation requires large biobanks, standardized outcome definitions, ancestry-aware interpretation, and external validation.

Gut-brain axis-related signals have also become an emerging area of interest. In patients undergoing off-pump coronary artery bypass grafting, alterations in gut microbiota diversity and short-chain fatty acid levels have been observed in patients who develop postoperative delirium, suggesting that microbial ecology and microbial metabolites may be linked to neuroinflammatory vulnerability (Huang et al., 2025). In a prospective matched multi-omic study of adults undergoing elective on-pump cardiac surgery, patients who developed postoperative delirium showed reduced preoperative microbial network connectivity and a postoperative microbiome–metabolome disturbance pattern centered on branched-chain amino-acid metabolism (Lyu et al., 2026). These studies remain observational and do not establish that microbiome manipulation prevents delirium.

Autonomic measures may reflect perioperative physiological regulation rather than brain injury itself. In a retrospective cohort of 71 older patients after cardiovascular surgery, postoperative delirium was associated with differences in heart-rate-variability indices measured before ICU discharge (Tsukakoshi et al., 2023). In cardiac surgery, however, heart rate variability must be interpreted cautiously because arrhythmias, β-blockers, pacing, heart failure, and vasoactive drugs can substantially affect autonomic measurements.

Overall, multidimensional biomarkers may help define biological subphenotypes after cardiac surgery, but their current evidence base is small, methodologically heterogeneous, and largely unvalidated. None has demonstrated sufficient incremental value over clinical variables for routine prediction or treatment selection. The major biomarkers investigated in cardiac surgery-associated PND, together with their sample types, sampling windows, clinical endpoints, potential relevance, and translational limitations, are summarized in Table 1.

**TABLE 1:** Major biomarkers investigated in perioperative neurocognitive disorders after cardiac surgery: clinical evidence, potential relevance, and translational limitations.

Category	Biomarker	Cardiac surgery setting	Sample size	Sample and timing	Neurocognitive endpoint	Main evidence	Current clinical relevance	Key limitations	References
Neuronal and axonal injury	NfL	On-pump CABG and/or valve surgery; mixed cardiac-surgery cohorts	Brown et al.: *n* = 175; Wiberg et al.: *n* = 168 (47 POCD at discharge); Alifier et al.: *n* = 25	Plasma/serum; baseline, POD 1, 24 h, 48 h, discharge/POD 7	POD and delirium severity; POCD at discharge; 3-month cognitive outcome	Higher baseline NfL, but not the rise to POD, was associated with delirium in the nested cohort. In Wiberg et al., discharge NfL showed modest discrimination for POCD (AUC 0.64), while no group difference was found across repeated levels.	One of the most promising markers for preoperative vulnerability assessment and serial monitoring of evolving axonal injury.	No validated cardiac-surgery cutoff; delayed kinetics; modest discrimination; renal function, age, assay and endpoint heterogeneity; incremental value remains uncertain.	Alifier et al., 2020; Wiberg et al., 2022; Brown et al., 2024
	Total tau / p-tau181	Major or mixed cardiac surgery	Wiberg et al.: *n* = 168 (47 POCD); McKay et al.: 38	Serum/plasma; baseline, 24 h, 48 h, discharge; early postoperative sampling in delirium study	Delirium; postoperative neurocognitive decline and recovery trajectories	In Wiberg et al., postoperative tau was higher in patients with POCD and showed only modest discrimination (AUC about 0.63). McKay et al. reported postoperative elevations of total tau, p-tau181 and p-tau217 in a single-center case-control analysis.	Process-oriented indicator of acute neuronal stress or injury; possible component of a multimarker panel.	Small or case-control datasets; limited cardiac-surgery replication; perioperative tau elevation is not specific for Alzheimer pathology; no external validation.	Wiberg et al., 2022; McKay et al., 2022
	NSE	CABG and mixed cardiac surgery, often with CPB	Silva et al.: *n* = 88; Wiberg et al.: *n* = 168	Serum; preoperative, after induction, end of surgery, 6 h, 24 h; baseline, 24 h, 48 h and discharge	POCD at 21 and 180 days; POCD at discharge	Silva et al. found S100β more informative than NSE for POCD after CABG. In Wiberg et al., postoperative NSE was not associated with POCD at discharge.	Auxiliary marker of neuronal cytoplasmic injury; primarily useful for mechanistic interpretation.	Haemolysis and transfusion interference; extracerebral sources; inconsistent findings; limited neurological specificity.	Silva et al., 2016; Wiberg et al., 2022; Hassani Ahangar et al., 2025
Glial response and BBB dysfunction	GFAP	CPB cardiac surgery; off-pump CABG in one panel study	Reinsfelt et al.: *n* = 10; Wiberg et al.: *n* = 168; Szwed et al.: *n* = 30	CSF/serum before and after CPB; serum at baseline, 24 h, 48 h and discharge; panel sampling around off-pump CABG	Cognitive impairment at discharge; early postoperative cognitive dysfunction	GFAP rose after CPB with evidence of BBB disruption. In Wiberg et al., postoperative GFAP was higher with POCD but discrimination remained modest (AUC up to 0.64). The three-marker Szwed panel showed high apparent performance in only 30 patients.	Promising marker of astrocytic injury; likely more useful in a multimodal brain-injury panel than alone.	Modest standalone performance; small cohorts; panel overfitting; lack of validated cutoffs and external validation.	Reinsfelt et al., 2012; Wiberg et al., 2022; Szwed et al., 2021
	S100β	CABG and mixed cardiac surgery, frequently with CPB	Silva et al.: *n* = 88; systematic review/meta-analysis sample varies by included study	Serum; preoperative, after induction, end of surgery, 6 h and 24 h	POCD at 21 and 180 days	In the prospective CABG cohort, postoperative S100β was associated with later cognitive dysfunction and performed better than NSE, but results across cardiac-surgery studies remain heterogeneous.	Research marker of astroglial injury and BBB permeability; possible adjunct in early injury panels.	Strong time dependence; extracerebral release after surgical trauma; variable assays and thresholds; limited brain specificity.	Silva et al., 2016; Hassani Ahangar et al., 2025
	CSF/serum albumin ratio	Cardiac surgery with CPB	Reinsfelt et al.: *n* = 10	Paired CSF and serum; before and after CPB	Biological evidence of BBB disruption; neurocognitive outcomes not consistently established	The CSF/serum albumin ratio increased after CPB together with CSF glial and inflammatory markers, supporting perioperative BBB dysfunction.	Pathophysiologicall y direct measure of BBB integrity and useful mechanistic endpoint.	Requires paired CSF and blood sampling; lumbar puncture limits feasibility; small mechanistic cohort; no direct clinical cutoff.	Reinsfelt et al., 2012
Systemic and neuroinflammation	IL-6	Mixed surgical populations in meta-analysis; cardiac-surgery cohort in trajectory analysis	Peng et al.: meta-analysis across surgical populations;	Serum/plasma; serial perioperative and postoperative measurements	POD and postoperative cognitive decline	Higher perioperative IL-6 is associated with postoperative cognitive decline across heterogeneous surgical studies. Serial inflammatory trajectories may be more informative than a single measurement.	Readily interpretable marker of inflammatory burden; potential component of serial multimarker models or pharmacodynamic studies.	Not cardiac-surgery specific in the meta-analysis; low neurological specificity; confounding by infection and surgical burden; trajectory study available as abstract.	Peng et al., 2013
	IL-8 and CSF cytokine profile	Thoracic aortic surgery	Pereira et al: *n* = 10	CSF; perioperative/post operative sampling	Central inflammatory activation; specific independent neurocognitive prediction not established	Thoracic aortic surgery produced postoperative changes in CSF IL-8 and other cytokines, demonstrating central inflammatory activation without establishing independent prediction of a neurocognitive endpoint.	Mechanistic indicator of central inflammatory response; potential research endpoint.	Invasive CSF sampling; procedure-specific cohort; exploratory cytokine panel; no validated predictive model.	Pereira et al., 2022
	CRP	On-pump cardiac surgery in older adults; planned isolated CABG	Németh et al.: *n* = 42; Kotfis et al.: *n* = 968 (POD, *n* = 129)	Serum; Németh et al.: POD 1, 3 and 5; Kotfis et al.: preoperative and POD 1, 3 and 5	Early postoperative cognitive decline assessed with a neuropsychologic al battery at POD 7; POD screened twice daily with CAM-ICU during the first 6 postoperative days	CRP increased markedly after cardiac surgery but did not differ between high- and low-inflammatory-response groups or show a direct relationship with early cognitive decline in the 42-patient prospective cohort. In the 968-patient CABG cohort, CRP was higher preoperatively and on POD 5 in patients with POD, but not on POD 1 or 3, and it was not retained in the final independent prediction model.	Routinely available marker of nonspecific systemic inflammatory burden; potentially useful as an auxiliary variable or serial measure within multimarker models, but not established as a standalone predictor.	Not brain-specific; strongly affected by surgical trauma, infection, comorbidity and sampling time. Evidence is inconsistent across time points; no validated cardiac-surgery-specific cutoff or independent incremental predictive value.	Németh et al., 2017; Kotfis et al., 2019
	NLRP3 / caspase-1 / IL-1β / IL-18	Older patients undergoing cardiac surgery	*n* = 81	Serum; preoperative and perioperative samples including end of surgery	Early PND	NLRP3, caspase-1, IL-1β and IL-18 increased after cardiac surgery; end-of-surgery NLRP3 was independently associated with early PND in this single-center observational cohort.	Mechanistically specific candidate for inflammatory phenotyping and pathway-engagement studies.	Single-center observational study; modest sample; no external validation; assay and cutoff standardization lacking.	Ma et al., 2022
Oxidative stress, neurotrophic and endogenous protection	BDNF	Elective on-pump CABG; CABG cohort	*n* = 45	Serum; baseline and intraoperative samples including aortic cross-clamping; serial postoperative follow-up	Early PND or postoperative cognitive decline	In a 45-patient pilot study, lower intraoperative BDNF during CPB/aortic cross-clamping was associated with early PND. Additional observational data linked persistent BDNF reduction with inflammatory and oxidative abnormalities.	Marker of impaired neurotrophic support or reparative capacity; candidate for mechanistic stratification.	Pilot sample; uncertain assay threshold; unclear independent and incremental predictive value; limited replication.	Miniksar et al., 2021
	HMOX1 / LOX-1	CABG	Miceli et al.: *n* = 20 (POCD = 9)	Circulating blood; perioperative and follow-up measurements	Postoperative cognitive decline	Lower HMOX1 and higher LOX-1 during follow-up were reported in CABG patients with postoperative cognitive decline, suggesting insufficient antioxidant defense and persistent oxidative-inflammatory stress.	Mechanistic phenotype of impaired endogenous defense and oxidative stress.	Single cohort; exact timing and predictive metrics require full-text confirmation; no external validation or clinical threshold.	Miceli et al., 2025
	SIRT1	Older patients undergoing CPB cardiac surgery with aortic cross-clamping	Shi et al., 2024 : *n* = 285 (POCD = 109); Shi et al., 2020: experimental model	Serum; early postoperative sampling	Postoperative cognitive dysfunction	Lower early postoperative serum SIRT1 was associated with cognitive dysfunction and inversely related to NSE/S100β. Experimental SIRT1 activation attenuated TLR4/NF-κB signaling and cognitive deficits.	Mechanistic stratification and pharmacodynamic research endpoint; not a validated clinical predictor.	Human evidence from a single observational cohort; substantial preclinical component; no validated cutoff or intervention trial.	Shi et al., 2024; Shi et al., 2020
Metabolomic and lipidomic biomarkers	Plasma lipidomic signatures	Cardiac surgery with CPB	Chen et al.: *n* = 42 (DNR = 21)	Plasma; early postoperative sampling	DNR after cardiac surgery	Early postoperative lipidomic signatures involving phosphatidylinositol, glycerophospholipid and sphingomyelin pathways differentiated patients with and without DNR in an exploratory study.	Exploratory postoperative risk classification and mechanistic phenotyping.	High-dimensional feature selection; small discovery cohort; platform dependence; overfitting risk; external validation absent.	Chen et al., 2023
	iPLA2 / SPT and hippocamp al lipid distribution	Rat CPB model	Rat CPB model	Hippocampal tissue; after experimental CPB	Postoperative cognitive dysfunction-like behavior	Spatial metabolomics showed reduced hippocampal iPLA2, increased SPT, altered lipid distribution and synaptic injury; DHA and myriocin partly reversed metabolic and behavioral abnormalities.	Preclinical mechanistic and target-engagement evidence only.	Animal tissue findings are not equivalent to a clinically measurable circulating biomarker; human replication and peripheral-central concordance are lacking.	Lei et al., 2024
Peripheral cholinergic biomarkers	AChE / BChE	Mixed cardiac surgery	*n* = 114	Whole blood or plasma; preoperative and postoperative measurements	POD	Lower preoperative AChE activity was independently associated with POD, and patients who developed delirium showed a greater perioperative decline in BChE.	Exploratory indicator of cholinergic vulnerability or systemic reserve.	Single-center cohort; peripheral enzyme activity may reflect systemic reserve rather than central cholinergic function; findings inconsistent across studies.	Adam et al., 2020
	BChE change	Elective on-pump CABG	*n* = 93	Whole blood/serum enzyme activity; baseline and POD 1	POD	The reduction in BChE from baseline to POD 1 was greater in patients with POD; a decline of at least 32% showed only moderate apparent discrimination and was not externally validated.	Potential inexpensive auxiliary marker for early POD risk.	Single-center study; moderate discrimination; data-derived threshold; no external validation.	Zajonz et al., 2023
	BChE	Cardiothoracic surgery and percutaneous valve replacement	*n* = 237 (POD = 94)	Blood; perioperative sampling	POD	Lower perioperative BChE was associated with POD in unadjusted analyses, but the relationship weakened after adjustment for age, comorbidity, procedure type and transfusion.	Possible contextual marker of systemic reserve rather than brain-specific injury.	Mixed procedures; residual confounding; attenuated adjusted association; limited brain specificity.	Schlake et al., 2024
Emerging multidimensional biomarkers	Circulating EV profiles	Cardiac surgery; other cardiac surgery with CPB	Wang et al.: *n* = 35 (POCD = 12)	Plasma exosomes; blood collected 1 day before surgery and POD 7	General postoperative complications; neurological/PND endpoints not consistently isolated	Exosomal circRNA_089763 was higher in patients with POCD after CABG, but the finding was derived from a very small discovery and validation sample. The broader cardiac-surgery literature summarized by	Exploratory molecular phenotyping and mechanistic research; a potential component of future multimarker models, but not established for routine PND risk prediction	Variable isolation, normalization and cargo assays; heterogeneous endpoints; limited direct PND evidence.	Wang et al., 2019
	miR-21-5p + GFAP + neuroserpi n	Elective off-pump CABG	*n* = 30	Circulating blood; perioperative/earl y postoperative sampling	Early postoperative cognitive dysfunction	A combined miR-21-5p, GFAP and neuroserpin model showed high apparent discrimination for early cognitive dysfunction, but the dataset contained only 30 patients and lacked external validation.	Promising multimarker discovery signal integrating regulatory RNA and brain-injury proteins.	Very small sample and event count; high overfitting risk; preanalytical miRNA variability; no external validation.	Szwed et al., 2021
	lncRNA-MYL2-2 / miR-124-3p	Cardiac surgery	Clinical cohort *n* = 450; biomarker substudy *n* = 29 (PND *n* = 10,non-PND *n* = 19)	Serum; perioperative sampling	PND or postoperative cognitive decline	Higher lncRNA-MYL2-2 and lower miR-124-3p were reported in association with perioperative neurocognitive disorders, suggesting a regulatory RNA signature.	Exploratory regulatory signature for phenotype discovery.	Single discovery cohort; reference gene and haemolysis sensitivity; incomplete external validation; exact effect estimates require full-text verification.	Xiao et al., 2021
	Gut microbiota and short-chain fatty acids	Off-pump CABG and elective on-pump cardiac surgery	Huang et al.: Human *n* = 120; Mice = 24; Lyu et al.: Prospective cohort *n* = 317; matched multi-omic cohort *n* = 60 (POD *n* = 30,non-POD *n* = 30)	Feces and microbial metabolites; perioperative serial sampling	POD	POD was associated with reduced microbial diversity, depletion of short-chain-fatty-acid-producing taxa and lower fecal SCFAs; animal transfer experiments supported biological plausibility. Multi-omic findings remain observational.	Exploratory vulnerability and systems-phenotyping signal; potential basis for future enrichment studies.	Strong confounding by antibiotics, fasting, opioids, nutrition and ICU exposure; center and geography dependence; no evidence that microbiome manipulation prevents POD.	Huang et al., 2025; Lyu et al., 2026
	Heart-rate variability	Older patients after cardiovascular surgery	Tsukakoshi et al.: *n* = 71	Physiological signal; measured before ICU discharge	POD	Postoperative HRV indices before ICU discharge differed in older cardiovascular-surgery patients with POD; exploratory work also linked preoperative autonomic tone with postoperative inflammatory trajectories.	Adjunct physiological phenotype rather than a molecular brain-injury biomarker.	Retrospective/sm all cohorts; arrhythmia, pacing, beta-blockers, heart failure and vasoactive drugs strongly confound HRV.	Tsukakoshi et al., 2023

AChE, acetylcholinesterase; BBB, blood-brain barrier; BChE, butyrylcholinesterase; CABG, coronary artery bypass grafting; CPB, cardiopulmonary bypass; DNR, delayed neurocognitive recovery; EVs, extracellular vesicles; GFAP, glial fibrillary acidic protein; HMOX1, haem oxygenase-1; HRV, heart-rate variability; NfL, neurofilament light chain; NSE, neuron-specific enolase; PND, perioperative neurocognitive disorders; POD, postoperative delirium; SCFA, short-chain fatty acid; SIRT1, sirtuin 1.

## 3 Biomarker-informed intervention strategies and translational gaps

Associations between biomarkers and PND have not yet translated into routine treatment selection. At most, a biomarker may identify a high-risk subgroup, define a biological phenotype, indicate when injury is evolving, or show whether an experimental intervention has affected its intended pathway. This is biomarker-informed care, not biomarker-guided therapy.

### 3.1 Biomarkers for identifying intervention windows rather than prescribing treatment

Inflammation- and BBB-related biomarkers may help identify biologically vulnerable periods, but they are not yet sufficient to directly guide standardized clinical decisions. The key issue is not simply whether inflammatory markers increase after surgery, but whether the inflammatory response is excessive, persistent, poorly resolved, or accompanied by evidence of central neurovascular or glial injury. Persistently elevated IL-6 may indicate unresolved postoperative inflammation, whereas elevated GFAP and an increased CSF/serum albumin ratio may suggest BBB disruption and central reactive injury (Peng et al., 2013; Reinsfelt et al., 2012). NLRP3 inflammasome-related markers measures are mechanistically more specific, but the available observational evidence does not justify targeted treatment in cardiac surgery patients (Ma et al., 2022).

Importantly, these biomarkers should not be interpreted as indicators for a specific drug or single intervention. Their more practical value is to identify patients who may have entered a biologically vulnerable phase in which standard perioperative protective measures should be intensified. Such measures may include optimization of cerebral perfusion, avoidance of excessive sedative exposure, correction of hypoxemia and anemia, infection control, sleep and pain management, early mobilization, and multicomponent delirium prevention. In this context, biomarkers provide a basis for stratifying perioperative care rather than prescribing treatment on their own (Majewski et al., 2020; Evered L. A. et al., 2022; Ran et al., 2025). Future studies should therefore test whether biomarker-defined injury patterns can identify patients who benefit most from intensified perioperative brain-protection bundles, rather than assuming that all high-risk patients require the same preventive strategy.

### 3.2 Biomarkers as pharmacodynamic readouts in pathway-targeted studies

Inflammation-related biomarkers may also serve as pharmacodynamic readouts in pathway-targeted intervention studies. Current evidence is derived mainly from experimental models, but it illustrates how biomarkers can move beyond risk prediction and help determine whether an intervention truly modifies the intended biological process. In an aged mouse model, sodium butyrate reduced IL-1β, IL-6, and TNF-α levels while improving postoperative delirium-like behavior, suggesting that inflammatory markers can be used to assess whether inflammatory amplification has been suppressed (Xu et al., 2024).

However, current evidence primarily supports the intervenability of inflammatory pathways in experimental settings and does not yet demonstrate that these biomarkers can reliably monitor clinical efficacy. Future trials could prespecify inflammatory, inflammasome, oxidative, neurotrophic, metabolic, or resolution-related markers as secondary or mechanistic outcomes rather than using them post hoc. This would allow investigators to distinguish biological non-response from clinical non-response and to refine subsequent trial design.

### 3.3 Matching biomarkers to clinical purpose and timing

The translational value of a biomarker after cardiac surgery depends on when it is measured and what clinical question it is intended to address. Preoperative markers are most relevant to vulnerability assessment. For example, higher baseline NfL has been associated with poorer preoperative cognition and a greater risk of postoperative delirium, whereas its early postoperative change has shown limited additional association with delirium in several cardiac-surgery cohorts (Brown et al., 2024; Khalifa et al., 2024). These findings illustrate that the same biomarker may reflect pre-existing cerebral vulnerability at baseline but acute neuroaxonal injury when measured serially after surgery.

A major translational issue is that different biomarkers are informative at different perioperative phases. Preoperative biomarkers may be more useful for identifying vulnerability and selecting patients for preventive strategies. Intraoperative or immediate postoperative biomarkers may be better suited to detecting acute biological injury and adjusting perioperative management. Delayed postoperative biomarkers may be more informative for monitoring ongoing axonal injury, unresolved inflammation, or failure of recovery. Without matching biomarker timing to the intended clinical purpose, even biologically plausible markers may show weak or inconsistent predictive performance.

Within this framework, SIRT1 and other endogenous protective markers may be useful, but their role remains preliminary. Lower early postoperative SIRT1 has been associated with cognitive impairment after CPB cardiac surgery, suggesting possible relevance to postoperative stress responses rather than established predictive utility (Shi et al., 2020, 2024). Such markers should be incorporated into cardiac-surgery studies only when the sampling schedule, proposed mechanism, and neurocognitive endpoint are prespecified. Their value will depend on whether they improve risk classification, demonstrate pathway engagement, or track recovery beyond routine clinical variables and established injury markers.

### 3.4 Biomarker-enriched trial design for metabolic and multi-omics interventions

Early postoperative lipidomic profiles have been associated with delayed neurocognitive recovery after cardiac surgery with cardiopulmonary bypass, but the reported signatures remain exploratory and require external validation (Chen et al., 2023). Preclinical evidence from a CPB rat model has shown that decreased hippocampal iPLA2 and increased SPT are accompanied by cognitive impairment, and that intervention with docosahexaenoic acid and myriocin can partially reverse these metabolic abnormalities and reduce postoperative cognitive dysfunction (Lei et al., 2024). These findings suggest that lipid metabolic remodeling may represent a modifiable pathway.

However, this area remains largely preclinical. Key translation will require trials that use metabolic biomarkers for a defined purpose. Preoperative or early postoperative multi-omics profiles could enrich enrolment for patients with the pathway abnormality targeted by the intervention, while serial measurements could test whether that abnormality is modified during treatment.. Future studies should therefore determine whether peripheral signatures reflect cerebral metabolic changes, remain reproducible across operative and analytical conditions, and add value beyond conventional clinical risk factors. Cognitive outcomes must remain the efficacy endpoint; metabolomic changes should be interpreted as evidence of subgroup selection or pathway engagement rather than as proof of clinical benefit.

### 3.5 Intercellular communication and gut-brain axis-related intervention strategies

EVs, microRNAs, and gut-brain axis-related signals provide a broader systems-level window into PND pathophysiology and expand the scope of potential interventions (Carrozzo et al., 2025). However, the immediate challenge is methodological reproducibility rather than therapeutic application

For EVs, it is important to distinguish EVs as biomarkers from EVs as therapeutic agents. Biomarker studies require standardized isolation, normalization, vesicle characterization, and cargo analysis, whereas therapeutic studies require rigorous control of source, dose, biodistribution, manufacturing reproducibility, and safety (Shao et al., 2018; Greening et al., 2025). Similarly, microRNA-based approaches require careful distinction between their role as circulating regulatory signatures and their potential role as therapeutic targets or agents. Without standardized analytical platforms and validated disease-specific signatures, these approaches remain more useful for phenotype discovery than for immediate clinical decision-making.

Gut-brain axis-related interventions face a different set of challenges. Perioperative confounders such as antibiotics, fasting, opioids, proton pump inhibitors, nutritional changes, infection, and ICU exposure can strongly influence microbiota composition and microbial metabolites. These factors should be incorporated into study design rather than treated as background noise (Mayer et al., 2022). At present, gut-brain axis-related biomarkers and interventions remain in the exploratory stage, and a stable clinical translation pathway has not yet been established. These fields should therefore be framed as platforms for phenotype discovery and intervention hypothesis generation rather than as near-term clinical tools.

### 3.6 Translational priorities and prospective validation framework

Current research suggests that biomarker studies are gradually moving beyond risk prediction toward intervention target identification and treatment-response assessment. However, the translational challenge in biomarker research for PND after cardiac surgery is no longer simply the lack of candidate biomarkers, but the absence of studies that link reproducible biomarker measurements to clearly defined clinical decisions. Most current evidence remains derived from exploratory cohorts or preclinical models, and true biomarker-guided intervention is still rare. Therefore, future research should shift from broad association testing toward purpose-driven biomarker validation.

A useful biomarker study should prespecify whether the marker is intended for preoperative risk enrichment, detection of acute neurological injury, identification of unresolved pathology, prognostic assessment, or evaluation of treatment response. Patient selection, sampling schedule, assay platform, outcome endpoint, and proposed management action should all be aligned with this intended use. For example, prospective studies may test whether a biomarker panel improves POD risk stratification beyond established clinical models, or whether serial measurements can detect evolving neurological injury after CPB. Prospective validation should be conducted in well-defined cardiac surgical populations with standardized biospecimen collection, assay procedures, and outcome assessment. Key perioperative variables, including CPB duration, aortic cross-clamp time, cerebral oxygenation, hemoglobin nadir, transfusion, anesthetic exposure, postoperative organ dysfunction, infection, and ICU treatment, should be recorded to account for cardiac surgery-specific confounding. Outcome assessment should distinguish POD, DNR, and postoperative mild or major neurocognitive disorder, using validated delirium instruments and harmonized cognitive testing with preoperative baseline assessment. Only through such purpose-driven validation can biomarkers move from mechanistic association toward clinically meaningful intervention frameworks. Finally, biomarker value should be judged by calibration, discrimination, incremental clinical utility, and external reproducibility rather than statistical significance alone. Biomarker-guided trials are warranted only when the assay is reliable, timely, biologically interpretable, and linked to a plausible change in perioperative management. Thus, the next step is not merely to discover more biomarkers, but to determine when, in whom, and for what clinical decision a biomarker can meaningfully improve perioperative brain protection.

## 4 Summary and outlook

Biomarkers research in cardiac surgery-associated PND has envolved from isolated candidate molecules to multimodal assessment, but no biomarker is currently ready for routine clinical implementation. This limitation reflects the biological and clinical heterogeneity of PND in cardiac surgical populations. Postoperative delirium, delayed neurocognitive recovery, and later postoperative neurocognitive disorder differ in timing, diagnostic approach, and likely biological correlates (Evered et al., 2018). In cardiac surgery, PND may arise from different combinations of pre-existing cerebral vulnerability, CPB-related injury, systemic inflammation, cerebral perfusion disturbance, BBB dysfunction, and postoperative recovery complications (Goto and Maekawa, 2014; Czok et al., 2021). Therefore, biomarker findings can only be meaningfully interpreted when the surgical context, sampling window, and neurocognitive endpoint are clearly defined.

Several methodological weaknesses continue to limit translation. Many studies remain small and single-center, use heterogeneous outcome definitions and sampling protocols, and derive biomarker cutoffs from the same cohorts in which performance is assessed. Assay variability and insufficient external validation are particularly problematic for multimarker panels and omics-based models. In addition, biomarkers are rarely evaluated against rigorous clinical reference models, despite the established prognostic contribution of age, frailty, baseline cognition, anemia, CPB exposure, hemodynamic instability, transfusion, renal dysfunction, and postoperative complications. Thus, clinical value should be defined not by statistical association alone, but by reproducible improvement in risk classification, monitoring, prognostic assessment, or management beyond these established variables.

Overall, NfL, GFAP, and selected inflammatory markers appear relatively closer to clinical translation because they are biologically linked to central nervous system injury and have been increasingly evaluated in perioperative settings. However, their interpretation remains highly time-dependent, and neither has a validated cardiac surgery-specific threshold. Inflammatory markers may be more useful as serial trajectories or components of multimarker panels than as isolated measurements, given their limited neurological specificity. By contrast, EVs, microRNAs, gut-brain axis-related signals, metabolomic profiles, and other emerging biomarkers remain largely exploratory because of limited standardization, platform heterogeneity, and insufficient validation. Current intervention evidence also suggests that modifying a single perioperative exposure is unlikely to prevent neurocognitive complications across an unselected cardiac surgical population. Biomarker-defined biological subgroups may offer a more rational basis for targeted prevention or treatment-response studies, but this approach remains unproven.

Ultimately, the most promising direction is unlikely to be a single universal biomarker, time-specific biomarker panels developed for defined clinical decisions represent a more realistic route toward translation. Progress will depend on purpose-driven biomarker frameworks that connect biological mechanisms with specific clinical decisions. The key question is no longer simply whether a biomarker is associated with PND, but whether it adds reproducible information, identifies a biologically meaningful or treatment-responsive subgroup, demonstrates pathway engagement, or supports a clinically actionable change in perioperative management. Multicenter validation should incorporate harmonized biospecimen procedures, standardized assays, cardiac surgery-specific exposure data, and appropriate clinical reference models. Future studies should prioritize harmonized outcome definitions, prespecified sampling schedules, standardized assays, external validation, and prospective testing of whether biomarker-defined phenotypes can improve patient-centered outcomes.
